# Challenges and opportunities in coproduction: reflections on working with young people to develop an intervention to prevent violence in informal settlements in South Africa

**DOI:** 10.1136/bmjgh-2022-011463

**Published:** 2023-03-29

**Authors:** Jenevieve Mannell, Laura Washington, Sivuyile Khaula, Zamakhoza Khoza, Smanga Mkhwanazi, Rochelle A. Burgess, Laura J. Brown, Rachel Jewkes, Nwabisa Shai, Samantha Willan, Andrew Gibbs

**Affiliations:** 1 Institute for Global Health, University College London, London, UK; 2 Project Empower, Durban, South Africa; 3 Gender and Health Research Unit, South African Medical Research Council, Pretoria, South Africa; 4 Department of Social Work, University of Johannesburg, Auckland Park, South Africa; 5 Office of the Executive Scientist, South African Medical Research Council, Pretoria, South Africa; 6 Psychology, University of Exeter, Exeter, UK

**Keywords:** Qualitative study, Prevention strategies, HIV

## Abstract

Coproduction is widely recognised as essential to the development of effective and sustainable complex health interventions. Through involving potential end users in the design of interventions, coproduction provides a means of challenging power relations and ensuring the intervention being implemented accurately reflects lived experiences. Yet, how do we ensure that coproduction delivers on this promise? What methods or techniques can we use to challenge power relations and ensure interventions are both more effective and sustainable in the longer term? To answer these questions, we openly reflect on the coproduction process used as part of Siyaphambili Youth (‘Youth Moving Forward’), a 3-year project to create an intervention to address the social contextual factors that create *syndemics* of health risks for young people living in informal settlements in KwaZulu-Natal province in South Africa. We identify four methods or techniques that may help improve the methodological practice of coproduction: (1) building trust through small group work with similar individuals, opportunities for distance from the research topic and mutual exchanges about lived experiences; (2) strengthening research capacity by involving end users in the interpretation of data and explaining research concepts in a way that is meaningful to them; (3) embracing conflicts that arise between researchers’ perspectives and those of people with lived experiences; and (4) challenging research epistemologies through creating spaces for constant reflection by the research team. These methods are not a magic chalice of codeveloping complex health interventions, but rather an invitation for a wider conversation that moves beyond a set of principles to interrogate what works in coproduction practice. In order to move the conversation forward, we suggest that coproduction needs to be seen as its own complex intervention, with research teams as potential beneficiaries.

**Summary box**

- Coproduction with potential end users has been widely accepted as good practice in developing and adapting global health interventions
- However, coproduction methods that lead to effective interventions remain poorly defined
- This article shares our experience codeveloping an intervention with young people in South Africa to explore the methods we used and their successes and challenges in practice
- Our study offers practical advice for researchers involved in codeveloping interventions, and highlights the need to approach coproduction as a transformative process for both researchers and participants alike

## Introduction

Increasing attention is being paid to coproduction—the involvement of potential end users in the design, delivery and evaluation of interventions—in the field of global health and medicine.^1^ The new National Institute for Health Research and UK Medical Research Council framework for developing and evaluating complex interventions highlight the importance of coproduction.^2^ Proponents suggest coproduction helps produce interventions that are more beneficial to end users,^3^ have improved impacts on health and well-being,^4^ are more ethical^5 6^ and are better able to reduce research waste.^7^ While coproduction can take time and substantial investment,^8^ it is now recognised as essential to the development of effective and sustainable interventions in health research.^9^

Coproduction is situated within a long legacy of interest in user involvement and participation in public health, public administration and international development, spanning decades.^10^ The term encapsulates a range of processes linked to codesign, cocreation and coevaluation, and different strands of debate have led to some murkiness around what actually counts as coproduction. On the one hand, coproduction is seen as the substantial involvement of citizens in public health decision making,^1^ while on the other, it is a transformative revisioning of how to conduct health research with marginalised groups.^11^ Whatever manifestation coproduction takes in practice, power inequities in voice, position and representation often pose a significant challenge to the equality of all parties involved in the process.^12 13^

In this article, we focus on the codevelopment of complex health interventions as a subset of coproduction. While detailed guidance does exist on developing partnerships with key stakeholder and service providers,^14^ how to develop a programme theory of change^15 16^ and steps for developing high-quality interventions,^17^ less attention has been paid to methods for collaboratively developing interventions in partnership with potential end users or the ‘how to’ of codevelopment as patient and public engagement in intervention design.^18^ The majority of attention has been on establishing key principles rather than developing specific methods and tools,^19 20^ critiquing the way interventions have been designed^21 22^ or conceptualising participation in research more broadly.^23–25^ As a result, a sizeable gap remains in the specific methods that can, and in some cases, should be used for doing codevelopment effectively.

To help address this gap, we argue for clear and explicit engagement in the methodological decision-making processes surrounding the codevelopment of interventions for complex social problems, such as the prevention of violence against women and girls, human trafficking, HIV and AIDS, and mental health in postconflict settings.^26–28^ The codevelopment process would not be exactly the same for these different interventions; however, an open methodological discussion about what works and what does not work can help generate new ideas for the practice of codeveloping complex interventions to improve health outcomes.

This methodological discussion is urgently needed for a number of reasons. While not the goal of all complex interventions, significant challenges exist in ensuring the meaningful engagement of potential end users in interventions that claim to be codeveloped. The extent to which power and control are shared with participants as part of intervention development is at best inconsistent and at worst superficial.^29^ Potential end users may be involved in intervention development as a member of the committee or research board but do not have any actual claim over how the research is being carried out.^21 25^ This type of superficial codevelopment is not neutral, but actually risks reproducing rather than challenging existing power inequalities in research relationships.^11 30^ Moreover, groups who are already socially and structurally marginalised tend to bear the burden of superficial codevelopment, including young people,^28 31^ those living with HIV/AIDS^32^ and women experiencing intimate partner violence (IPV).^32 33^

Despite a handful of thought-provoking efforts to describe case studies of codevelopment practice,^11–13 23^ the absence of a broader discussion has left a number of important questions unanswered, particularly around what is needed to codevelop better and more effective interventions. For instance, how does one avoid replicating and, where possible, challenge existing power hierarchies in research or overcome historical epistemic injustices? How can we find a balance between often competing perspectives such as end users’ lived experiences and the worldview of the researchers and perspectives gleaned from published literature? What methods can we use to ensure programme theories of change are grounded in end users’ experiences? We approach these questions from a critical public health perspective that draws attention to how structural inequalities shape and define social practices^34^ and use this to explore codevelopment as a *social practice* that is intimately intertwined with existing hierarchies of power and privilege.

To accomplish this aim, this article draws on our specific experience codeveloping an intervention to address the syndemics of IPV, HIV, mental health and harmful alcohol use affecting young people living in urban informal settlements in South Africa.^35^ We reflect on our experiences as part of this project and draw broader lessons for the field of global public health. The need to address the broader structural inequalities that underpin many of the challenges faced by young people in this context has been a tension throughout the project. Some of our strategies to overcome these challenges have succeeded, while others have failed. We discuss specific examples and summarise the methodological insights (ie, the theoretical assumption that underpin our selection and use of particular methods) for coproducing interventions in global health.

### The project: Siyaphambili Youth

Siyaphambili Youth (‘Youth Moving Forward’) is a 3-year research project aimed at addressing the social contextual factors that create a *syndemic* of high rates of IPV, HIV and poor mental health among young people living in informal settlements in KwaZulu-Natal (KZN) province in South Africa. The project is a partnership between the South African Medical Research Council (SAMRC), a South Africa non-governmental organisation (NGO) called Project Empower (PE), University College London (UCL) and a team of 17 young peer research assistants (YPRAs) living in urban informal settlements and rural communities in KZN. The principal investigators, coinvestigators and research staff from SAMRC, PE and UCL are referred to jointly as the ‘research team’ throughout this article as a means of discussing the relationship between the research team and the YPRAs as a central focus.

YPRAs were recruited to the project through an iterative hiring process designed to maximise possibilities for vulnerable youth to participate. Preliminary selection criteria included youth who were not currently in school or formal work, and were between 18 and 29 years old. The majority of those recruited had completed grade 12 but had not gone on to higher education. Three had stopped education after grade 11, and one had only completed grade 8. An informal introduction to the project was held in both rural and urban locations, in addition to proactive recruitment of young men by approaching them in places where they were seen to congregate. The project responsibilities, ethical considerations and payment terms were discussed in detail over the course of several days with potential YPRAs before they were asked to give their consent and were officially hired by PE. Following this process, five urban women, four urban men, four rural women and four rural men joined the Siyaphambili Youth Project as YPRAs.

**Figure 1:**
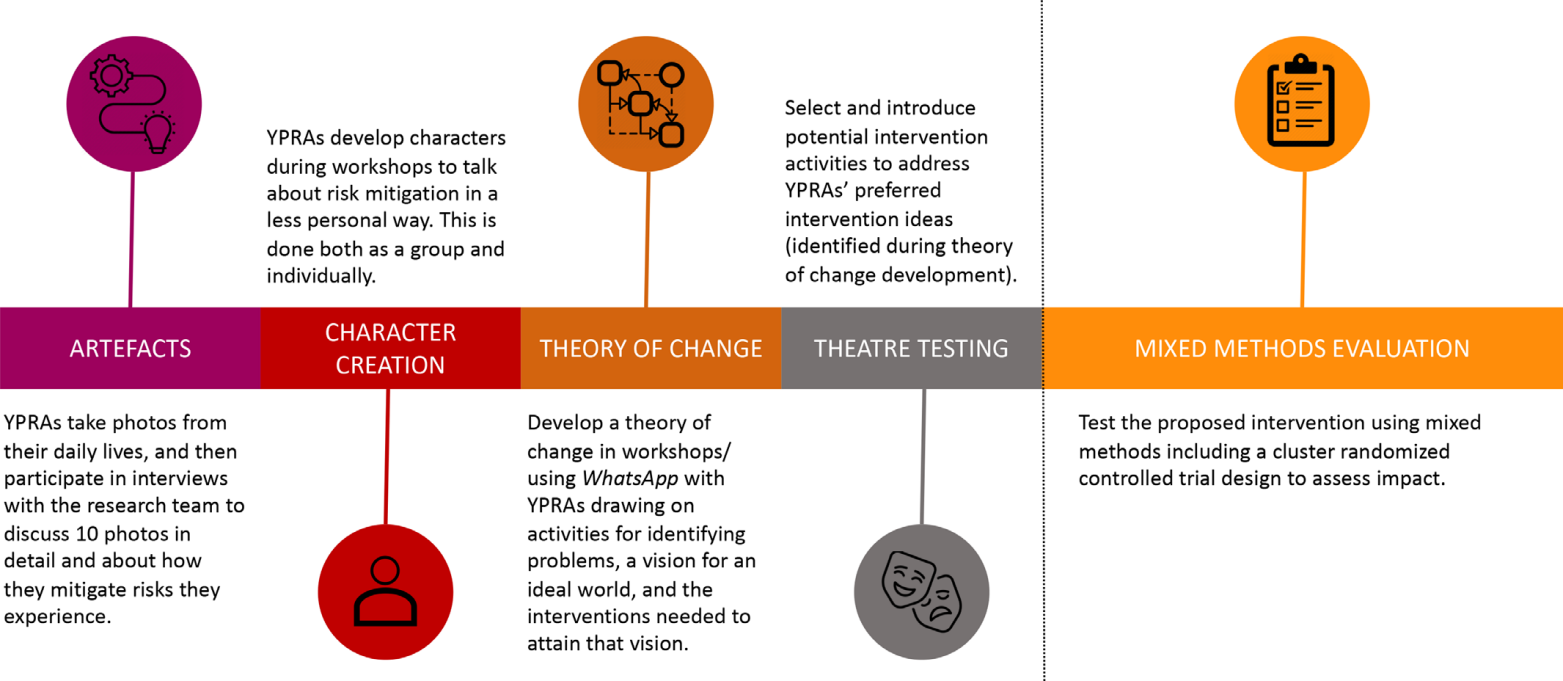
Siyaphambili youth intervention development study phases. YPRA, young peer research assistant.

Since November 2020, the YPRAs have participated in four phases of development of an intervention (figure 1). The first phase focused on understanding the syndemics of HIV, IPV and poor mental health risks (ie, how social contexts create overlapping risks characterised by poverty, violence, mental health vulnerabilities and gender inequalities), and young people’s agency in the face of such risks. YPRAs individually took photographs representing their daily lives (referred to as ‘artefacts’) and participated in in-depth interviews to discuss their meanings. In the second phase, YPRAs were involved in a series of workshops where they drew on their personal experiences to develop fictional characters, which were then used for facilitated discussions on young people’s daily lives.

The third phase built on the previous two phases to develop a theory of change for the intervention. YPRAs did this by identifying risk and protective factors for HIV and IPV as part of a problem tree participatory workshop activity. They were asked to visualise risk factors for HIV and IPV including the underlying causes (tree roots), the more proximal causes such as behaviours (tree trunk) and the effects on young people’s lives (tree branches). A similar tree was created for the ‘solutions’ or actions that YPRAs thought would prevent HIV and IPV. As part of this process, the research team shared analyses from other research activities conducted as part of the project with the YPRAs (ie, a quantitative analysis on alcohol use in similar communities, and qualitative analysis of artefact interviews with YPRAs) to explore new ideas or alternative ways of seeing the problem and its potential solutions. YPRAs were then asked to imagine an ideal world where the problems they had identified did not exist and to think of potential interventions that could help them to achieve this ideal world. During this activity, the YPRAs created visual maps outlining the problems, their intervention ideas and intended outcomes (ideal-world scenario). These maps were then shared with the broader research team to refine the intervention pathways based on existing evidence.

The fourth and final phase involved introducing the YPRAs to an evidence-based intervention to reduce HIV and IPV. The theory of change process pointed to the need for a gender transformative intervention with varying degrees of livelihood creation, mental health support and broader structural change for each of the four groups (men vs women and urban vs rural). We used the Stepping Stones and Creating Futures intervention,^36^ which has been shown to effectively reduce men’s perpetration of violence in the South African context,^37^ as a starting point. We adapted the original intervention to incorporate broader mental health support and to be more aligned with the theory of change developed with the YPRAs. The YPRAs were closely involved in adapting the overall content of the interventions and the individual activities to suit their context and unique needs as identified in the theory of change process.

### Patient and public engagement

The form of codevelopment discussed in this article (ie, codeveloping an intervention with potential end users) is a form of patient and public engagement that aims to engage the public in the research process itself. The ‘public’ in the Siyaphambili Youth project were the 17 YPRAs who were integrally involved in all four phases of the intervention development process as mentioned. In addition, YPRAs were involved in other aspects of the study, including recruitment of participants from their peer groups for in-depth qualitative research on topics such as rioting, emotional dysregulation and sexual life histories.

## Coproduction successes and failures

### Building trust: involving potential end users as researchers

The direct involvement of YPRAs provided opportunities for understanding their everyday lives, which was one of the main objectives of the project. This was greatly facilitated by both the length of the project (eg, being able to build up trust over several years instead of hours or days), and the use of participatory methods to open up different conversational starting points.

The use of YPRAs’ photographs as a starting point for an in-depth interview (ie, the artefact interviews) provided an opportunity to explore complex social dynamics, including the meaning of relationships, dating violence and how food insecurity plays out in young people’s lives. As a specific example, several of the young women shared photos of pizza, and it was only through later discussions with the research team that they explained its importance as a status symbol in their lives. One woman in an urban setting explained a personal strategy of posting pictures of pizza bought by her friends’ boyfriend on social media so that her own boyfriend would also feel pressure to provide. Another took photos of yoghurt, which was also a representation of the expectation that love is expressed through men providing ‘luxury’ food items for their girlfriends. A third photo was of a group of young men and women at the beach, which for the young woman was evidence of how she had had to invite her female friends to join her because she was worried about being alone with her boyfriend and his male friends.

The use of photo elicitation for interviews created a conversational starting point for discussions with the research team about the YPRAs’ everyday lives. However, the YPRAs were still hesitant to discuss aspects of their lives that they thought the research team might not understand, like drug use or violence in their relationships. As paid employees, the YPRAs were concerned about how illegal and negative behaviours would impact on their involvement in the project. YPRAs were equally cautious of sharing personal information with one another, and it took months for them to openly discuss their personal lives in a group.

The challenges related to YPRAs’ willingness to share personal information were overcome to a certain extent through constant interaction and trust-building exercises carried out by a small group of expert facilitators on the research team. These expert facilitators used a range of techniques, including formal methods such as community mapping, character creation and photography, as well as informal techniques to create a non-judgemental and trusting space. Informally, the facilitators achieved a sense of comradery and group belonging through sharing examples from their own personal lives and pulling the YPRAs aside for private group ‘chats’ away from the rest of the research team and the recording equipment. In addition to the participatory methods, these informal techniques were instrumental in building trust with and between the YPRAs over time.

While there were interruptions in the research schedule as a result of COVID-19 lockdown measures, flooding and an attempted insurrection, these also provided a means of extending the project timeline, which gave the research team additional time to build trust with the YPRAs. After 2 years of regular meetings (one to two per month) between the research team and the YPRAs (for 1 year more than originally planned), trust has been established to some degree, and more details about the YPRAs lives have come to the surface. However, like any employer–employee relationship, there continues to be a hesitation by the YPRAs in sharing the intimate details of their lives.

### Giving interpretive ownership over data

In trying to create a space for meaningful codevelopment, we selected methods to facilitate more open conversations about the YPRAs’ lived experiences, as well as strengthening capacity in understanding research and how it operates. The research team did this through introducing results from our own analyses during workshops with the YPRAs, particularly during the codevelopment of the theory of change. As part of this process, the research team explained key research concepts and discussed data interpretation. For example, differences between causation and correlation were raised in relation to COVID-19, with YPRAs discussing how 5G towers were said to be a key cause of the pandemic. The research team also introduced some key findings about the rates of violence in South Africa’s urban informal settlements.

These activities demonstrated that YPRAs understood the structural barriers that create disadvantage for themselves and their families, including barriers to engaging in South Africa’s democratic processes. They engaged with aspects of the data conceptually; for example, the rural YPRAs openly challenged how the research team had equated food insecurity and poverty in a previous study.^38^ The YPRAs asserted that poverty was not having the resources to lead an ideal life rather than just whether or not they went to bed hungry. They also openly disagreed with certain results, for example, they felt that the violence they had personally witnessed within their families was higher than that found in previous studies.

While the research team was excited about many of the insights the YPRAs brought to the table, there were also roadblocks in the YPRAs’ ability to engage in the research process. For example, the YPRAs found it challenging to develop fictional characters that were separate from themselves and their own stories, the fictional characters we had hoped would enable them to speak about issues without ‘exposing’ themselves to comments from others. Moreover, when asked to identify potential intervention ideas or strategies for achieving an ideal world, the young women from urban informal settlements in particular found it nearly impossible to come up with ideas. They had never imagined a life for themselves that was different from what they were currently living. While the coproduction process was aligned with YPRAs’ lived experiences in the case of urban men and the two rural groups, coproducing an intervention for the young women was challenging simply because they were unable to conceptualise potential solutions to the problems they were facing.

### Embracing conflict: uncomfortable conversations

The YPRAs openly disagreed with the research team at several points in the project, and at times, this was challenging for the research team. For example, the young men living in the rural area openly condoned sexual harassment, including rape, if women dressed or acted in a particular way. Similarly, the young women living in urban areas wanted to stop abortion in their communities as a priority and perceived the problem as women not conducting themselves in ‘appropriate’ or ‘dignified’ ways. Both of these viewpoints made it difficult for the research team to come to terms with the YPRAs’ perspectives because it went against the evidence for preventing HIV and IPV risk and the research team’s own personal and political views. The young rural men’s assumptions of gender roles and the need to control women’s lives posed significant challenges for their involvement in an intervention aiming to prevent HIV and IPV.

The research team tried to overcome these conflicts during the codevelopment process by revisiting activities multiple times from different angles and with different questions. For example, when first asked to identify solutions to HIV and IPV risk, a common solution suggested by YPRAs was rehabilitation centres for alcoholics. It was only through further conversations that YPRAs were able to reflect more on their own lived experiences and to identify more practical and reflective ideas. In developing a theory of change for the intervention (phase III), the research team redid the activities multiple times to explore different approaches to the task. In the last rendition of these activities, rather than asking the YPRAs what they thought the problem and solutions were to HIV and IPV, the team posed questions about potential solutions to the problems the YPRAs had themselves already identified (eg, pressure from others to provide financially when they cannot) and how these were linked to more structural issues such as poverty. This helped to shift the conversation away from individualised interventions that were unlikely to work (eg, institutionalising alcoholics into rehabilitation programmes) to strategies that were both more reflective about the social structures young people face and more pragmatic about what can be done to address them, for example, providing mental health support, helping to build healthy relationships, strengthening communications skills and targeting small enterprise development.

### Challenging research epistemologies

The conflict that arose during the coproduction process between YPRAs and the research team as described contributed to an increased reliance by the research team on their own knowledge of methodology and the evidence base as part of the coproduction process. This brought about many lengthy discussions about methods and epistemology (ie, our assumptions about what constitutes evidence in different research paradigms) by our multidisciplinarily team of social psychologists, public health researchers, economists, ethnographers, statisticians, qualitative researchers, intervention specialists and social epidemiologists.

In searching for the perfect method, these discussions often obscured attention to the components that were most important for the coproduction process to work as intended. For example, the research team had a lengthy discussion about the sample size required for data collected when data quality was likely far more impacted by the trust and ownership that YPRAs felt over the research process itself. The emphasis on methodology and evidence also made it difficult for the YPRAs to engage with the research team in the ways originally planned as part of the project. We had originally hoped that the YPRAs would join the monthly meetings with the broad research team. However, involving the YPRAs in what were essentially non-participatory online monthly meetings with a group of academics with mixed social, economic and racial identities would have further emphasised the social marginalisation that the YPRAs already felt in comparison to the research team. As the project progressed, we increasingly worried that their involvement in discussions about sample size and methodology would make the YPRAs feel less secure in the importance of their knowledge to the project and reify rather than break down existing relations of power. Instead, we structured meetings between the YPRAs and individual academics, which helped address these barriers and provided lively spaces of conversation but did not facilitate YPRAs’ full involvement in project activities.

One consequence of this division between the research team and the YPRAs was that these two groups often had different interpretations of the data and, therefore, different interpretations of what was most important to address as part of the intervention. For example, the young women living in urban informal settlements described how they often did laundry for their boyfriends and cooked them food. Some members of the research team interpreted this as an example of gendered social inequalities, whereby young women provide free labour to their male partners to allow them to in turn gain money through formal employment. This then led to the suggestion that interventions should address the gendered inequalities within relationships, have young women stand up to their boyfriends and assert their ability to support themselves through their own livelihood strategies. However, an alternative perspective, and the one that the young women themselves presented, is that the process of doing laundry for their boyfriends provided a means of expressing domesticity and love in a context where relationships are often fleeting, not monogamous and characterised by conflict. The practice of doing laundry was not perceived as exploitation but rather a means of overcoming the equally oppressive social expectation that their relationship would fail. In contrast to a gender transformative intervention, this led to the suggestion that women should be supported in nurturing their relationships as a means of providing stability and love in their everyday lives, rather than being forced into formal paid employment. This example illustrates how we sometimes get it wrong as researchers, even when working in close collaboration with potential end users on a topic of mutual interest.

## Moving forward: towards new methodological approaches

The evidence suggests that coproduction has the ability to create more user-friendly interventions and evaluations.^3 4^ We first engaged in a process of coproduction as part of the Siyaphambili Youth project to try and address the repeated failures of interventions to reduce the risk of IPV and HIV among young people in South Africa.^28^ As the project moves forward into piloting and evaluating the intervention in the coming years, we will know more about whether or not we have achieved this goal.

In the meantime, we have observed several strengths and some weaknesses of the codevelopment methods used. While these observations cannot tell us about the effectiveness of the final intervention, they do help deepen understanding of how coproduction works in practice and the added value of codeveloping interventions with potential end users over the use of high-quality formative research. We summarise these in table 1, including the formal methods and informal techniques used and described previously, and the positive and negative outcomes that we observed over the course of the project.

**Table 1:** Coproduction methods for Siyaphambili Youth

Principles	Formal methods	Techniques (informal methods)	Outcomes (both positive and negative)
Building trust in research partnerships	Small group work with similar individuals using participatory methods. Providing opportunities to describe sensitive research topics from a distance (character creation).	Mutual exchanges about lived experiences	YPRAs feel more comfortable sharing their lived experiences. Hesitancy in sharing some experiences (eg, drug use) with their employer (the research team).
Interpretive ownership	Sharing data analyses as part of codeveloping a theory of change	Engaging in discussions about research (eg, causation vs correlation)	Increased capacity to understand the objectives of research. YPRAs not being able to imagine an ideal world or identify solutions.
Embracing conflict	Multiple iterations of activities and repetition across the research process	Using end users’ own language to frame problems and identify solutions	YPRAs are able to arrive at solutions that are reflective and practical. Some YPRAs may not have the self-awareness to deliver a gender transformative intervention.
Challenging research epistemologies	Multidisciplinary research teams as a means of improving interventions	Constant reflection about whether meetings/interactions strengthen or weaken power relations.	The research team is not always right about the interventions that YPRAs want to see. Involvement of YPRAs in every discussion may reify existing power inequalities.

YPRA, young peer research assistant.

These observations from the coproduction process used as part of the Siyaphambili Youth project highlight the complexity of the practicalities of coproduction and its unpredictability as a process more broadly. None of the methods we used effectively challenged power inequities. As a key example, the challenges young urban women face in defining an ideal life for themselves were not something we could fully overcome as part of the project; it stems from broader structural realities, such as gendered notions of the ‘ideal’ woman as subservient and quiet, in addition to poor levels of education, poverty at home and lack of opportunities to get rewarding jobs and advance themselves.^39^ However, careful reflection throughout the coproduction process by all parties did appear to be integral to the potential for the process to be a success in other ways. The use of participatory methods—widely recognised to address a set of problems that people themselves define as important^18 30 40 41^—helped to build the trust and level of engagement needed for YPRAs to openly share their life challenges with the research team. More specifically, methods such as photo elicitation, community mapping and story creation helped to develop different understandings of young people’s daily lives than would have been achieved through in-depth interviews, which provided a new point of comparison and depth to the data.

Introducing activities iteratively and repetitively, developing capacity for the YPRAs to engage with research concepts and using their own words to discuss topics helped the YPRAs reflect about their own lived experience in a way that they had not done previously. Coproduction is not only a means to an end (ie, producing a more effective intervention) but also an intervention in and of itself. As a result of the increased confidence that the YPRAs gained through the coproduction process (and if the right individual was facilitating), they were more able to challenge the evidence, and ultimately the older and more educated members of the research team, as they weighed up proposed findings against their lived experiences in suggesting potential intervention options. The ideas of the YPRAs also changed over the course of the project, with several YPRAs openly challenging their previous conceptions about gender norms, HIV and mental health. A research process that does not allow for this shift in perceptions would have lost the insights gained through the YPRAs’ own process of self-discovery during the project and the potential this has for thinking about effective interventions for their peers. The process of intervention development has as much to gain from observing this process of individual change as it does in understanding potential beneficiary perspectives on what might work best in practice.

The iterative and repetitive nature of activities also played an important role in the reflective process of the research team. The research team constantly asked questions of the data and whether these were answering the research questions, particularly when it came to developing the theory of change. This required humility and acceptance that activities might need to be done again, but the repetition also gave the YPRAs increasing confidence in their own understanding of the project’s goals and built up high levels of trust between the research team and the YPRAs. This resonates with calls for ‘slow science’ as a form of research driven by curiosity in the social world rather than outputs and outcomes.^42^

However, doing justice to the benefits of iteration, repetition and reflection as methods for effective codevelopment may require more than just a change of pace. As others have also highlighted, a shift in thinking about what constitutes research evidence may also be required.^34 43^ The widely accepted hierarchy of evidence in global health research that places randomised controlled trials at its pinnacle requires expertise that people with lived experience are far less likely to have. It also requires interventions (and consequently their theories of change) to be fully developed before being evaluated.^44^ As a consequence, the evidence drawn from lived experience may be glossed over in pursuit of an intervention that can be evaluated and contributes to the evidence base. A codevelopment epistemology that centres lived experience in intervention design also needs those with lived experience to be central to defining what kind of evidence is needed for evaluating success.^45^ This includes defining what success looks like in the first place, what outcomes should be measured and how to measure these outcomes.

Building on this need for a different epistemological approach to producing research evidence, we found that perhaps the most important methodological insight arising from the project is that coproduction is an intervention not only for the potential end users but also for the researchers involved. Madden and colleagues^18^ allude to this in suggesting that coproduction adds a new layer of complexity to the design of complex interventions beyond differential relations of power. In their work, Burgess and Choudary^12^ illuminated that within successful coproduction endeavours (defined as those which recognise and transfer power), change must also occur within the actors who traditionally hold decision-making power in health-related settings. Without such transitions, coproduction has been presented as an impossibility.^13^ The researchers involved in the coproduction process must be prepared to re-evaluate their own assumptions about how and why interventions work to make space for the alternative approaches that potential end users may bring to the table.

For this reason, conflict within coproduction projects is vital and not something to shy away from. Work by Mannell and colleagues in Samoa has demonstrated that this can often lead to more productive and indigenous understandings of key research concepts, including theories of change.^46^ As a methodological strategy, Chadwick^47^ suggests the need to engage with our ‘gut feelings’ and ‘interpretive hesitancy’ as part of the research process and to help explore alternative explanations for concepts we may take for granted. As a process seeking to bring together alternative perspectives with the aim of producing more effective interventions, providing space for different interpretations of the world provides a useful starting point. However, as raised by Staley,^4^ the benefits of coproduction are unpredictable at the outset, and while particular methods can potentially help improve the understanding of how context influences the mechanisms of an intervention, there is no guarantee that they will improve its effectiveness.

## Conclusions

Coproduction requires a shift in power and ‘expertise’, which is an uncomfortable position for many researchers.^48^ However, this is only the beginning of the challenges that coproduction raises for research practice. The coproduction process provides not only an opportunity for researchers to listen to what potential end users want from the interventions that affect their lives but also a fundamental shift in research practices and epistemologies. We have drawn on our example of the Siyaphambili Youth project to highlight the potential need for new methodological approaches that help to overcome some of these challenges, including through the use of participatory methods that are appropriately aligned with research objectives, sufficient time for multiple iterations of key processes and the creation of spaces for researchers to reflect on their own preconceptions about how and why interventions work. We hope that this will start a conversation with others about the empirical challenges and strategies for coproducing more effective interventions for global health and medicine.

## Data Availability

No data are available. Not applicable.
